# Evaluation of Thyroid Function Tests in Children with Chronic Liver Diseases

**DOI:** 10.4274/jcrpe.galenos.2019.2019.0029

**Published:** 2020-06-03

**Authors:** Ş. Şebnem Ön, Sezer Acar, Korcan Demir, Ayhan Abacı, Yeşim Öztürk, Sinem Kahveci Çelik, Ece Böber

**Affiliations:** 1 Dokuz Eylül University Faculty of Medicine, Department of Pediatrics, İzmir, Turkey; 2 Dokuz Eylül University Faculty of Medicine, Department of Pediatric Endocrinology, İzmir, Turkey; 3 Dokuz Eylül University Faculty of Medicine, Department of Pediatric Gastroenterology, İzmir, Turkey

**Keywords:** Pediatric or childhood chronic liver diseases, thyroid function test, euthyroid sick syndrome, subclinical hypothyroidism

## Abstract

**Objective:**

Studies examining changes in thyroid function in the course of chronic liver disease have mostly been conducted in adults. The aim of this study was to investigate thyroid dysfunction in children with chronic liver diseases.

**Methods:**

Between 2005 and 2018, patients aged up to 18 years of age, diagnosed with chronic liver disease and had thyroid function test results available were included. Anthropometric characteristics, liver and thyroid function results were collected and analyzed.

**Results:**

The study included 107 (53 female; 49.5%) patients aged between one month and 18 years-old. Of the 107 patients, 96 (89.7%) had normal thyroid function results, seven (6.5%) had subclinical hypothyroidism (SH) and four (3.7%) had euthyroid sick syndrome. Of the patients with SH, one (14.2%) had glycogen storage diasease, one (14.2%) had biliary atresia, one (14.2%) had undiagnosed cholestatic liver disease, one (14.2%) had Alagille syndrome, one (14.2%) had idiopatic hepatitis, one (14.2%) had progressive familial intra-hepatic cholestasis and one (14.2%) had congenital hepatic fibrosis. Spearman correlation analysis showed a negative correlation between free tri-iodothyronine and direct bilirubin (r=-0.329, p=0.027).

**Conclusion:**

In conclusion, euthyroid sick syndrome or SH may affect up to 10% of children with chronic liver diseases. It is suggested that thyroid function should be evaluated in cases of pediatric chronic liver disease at diagnosis and during follow-up. Moreover, this study is the first to show a negative correlation between free T3 levels and direct bilirubin, suggesting a possible association between liver disease severity and thyroid function.

**What is already known on this topic?**

Thyroid hormone metabolism may be impaired in adult chronic liver diseases and subclinical hypothyroidism (SH) (≈3.5%) or euthyroid sick syndrome (7-30%) may occur. There are very few studies in the pediatric age group investigating this issue.

**What this study adds?**

The proportion of pediatric/adolescent chronic liver disease patients with SH or euthyroid sick syndrome was around 10%. It should be kept in mind that children with chronic liver disease may have SH.

## Introduction

Thyroid hormone synthesis occurs in the thyroid gland and is mainly controlled by thyroid stimulating hormone (TSH) secreted by the anterior pituitary gland. The hormone that is mostly synthesized from the thyroid gland is tetraiodothyronine (T4), while the active form at the level of the cell is triiodothyronine (T3) (1). Iodothyronine seleno-deiodinase enzyme complex, which regulates thyroid hormone metabolism, consists of three types of enzymes: type 1, type 2 and type 3 deiodinase (2). Deiodineases are responsible for the conversion of T4 to T3 (active form), T4 to inactive reverse T3 (rT3) and the conversion of rT3 and T3 to diiodothyronine (T2) (2). T4, the main product of the thyroid gland, must be converted to T3 by type 1 or type 2 deiodinase enzymes in order to affect cell metabolism (1,2).

Studies have shown that chronic liver diseases can be associated with conditions indicating impaired thyroid hormone metabolism, including euthyroid sick syndrome and subclinical hypothyroidism (SH) (2,3,4,5,6). Euthyroid sick syndrome is a condition classically characterized by low serum T3, low or normal free T4 (fT4) and normal or low TSH (7). In a prospective study of 118 patients with cirrhosis, thyroid glandular volume increased by 17% compared to controls and moreover, low total or free T3 (fT3) and high rT3, suggestive of euthyroid sick syndrome, were demonstrated in thyroid hormone profiles (8). It has been suggested that this occurs as a result of a decrease in the activity of type 1 deiodinase and an increase in the activity of type 3 deiodinase (2). In a study of adult cases with acute and chronic liver diseases, the rate of thyroid dysfunction was found to be 16% and 7% of them were reported to have euthyroid sick syndrome (9). Caregaro et al (10) reported that the frequency of euthyroid sick syndrome to be 30.6% in a recent study of cirrhotic adult patients. SH is defined as a serum TSH level above the reference range with normal serum fT4 and fT3 levels (11). In the pediatric population, SH prevalence is reported to be slightly lower than 2%, although epidemiological studies concerning childhood and adolescence are scanty (12). In a study conducted with adult patients with acute and chronic liver diseases, SH was found in 3.5% of patients with thyroid dysfunction (9).

In the literature, there are only a few studies investigating the relationship between liver disease and thyroid dysfunction in the pediatric age group (13,14,15). In a study of children with glycogen storage disease (GSD), it was reported that fT4 levels were significantly lower in patients with GSD type 1a and type 1b and free T3 was reported to be significantly higher in patients with GSD type 1b than in the control group (13). In a study of children with cirrhosis it was reported that decreased levels of thyroid hormones correlated with the severity of disease, so that in more advanced cirrhosis, patients with decreased T4 concentrations were in imminent need of liver transplant although those with normal T4 concentrations, despite having severe cirrhosis, were able to delay liver transplant for longer (14). In another study of children with liver cirrhosis it was reported that fT3 concentrations were lower in patients than in controls (15).

It has been suggested that there is a relationship between hypothyroidism and autoimmune liver diseases, such as chronic active hepatitis and primary biliary cirrhosis (3). Thyroid dysfunction has been reported in 5% to 20% of patients with primary biliary cirrhosis and chronic active hepatitis, a frequency which is higher than reported for general population. There is insufficient data showing the relationship between childhood chronic liver disease and thyroid dysfunction. Therefore, the aim of this study was to identify thyroid dysfunction during the course of childhood chronic liver diseases and to contribute to the literature in order to better inform clinicians managing this group of patients.

## Methods

Between January 2005 and June 2018, pediatric and adolescent patients who were diagnosed with chronic liver disease by the pediatric gastroenterology and hepatology and nutrition departments of our hospital were included in this study. Chronic liver involves a process of progressive and irreversible damage in the liver, due to some acquired or congenital conditions, and subsequent regeneration of liver parenchyma leading to fibrosis and cirrhosis. Patients with acute hepatic disease, drug use that might impair thyroid function (such as dopamine or glucocorticoid) and known thyroid disease were excluded. The current study was approved by the Dokuz Eylül University Local Ethics Committee (protocol no: 2017/09-10) and was performed in accordance with the Helsinki Declaration.

Anthropometric data including age, gender, body weight, height, body mass index (BMI) and laboratory data including liver function test results for alanine aminotrasferase, aspartate aminotransferase, gamma glutamyl transferase, alkaline phosphatase, total bilirubin, direct bilirubin, indirect bilirubin, albumin, total protein, clotting studies which may be influenced by liver function (protrombin time, international normalized ratio) and thyroid function tests (TSH, fT4, fT3) were obtained from patient files. The BMI of each case was calculated by dividing weight (in kilograms) by the height (in squared meters). For calculation of percentiles and standard deviation scores (SDSs) of all anthropometrics according to Turkish child growth reference data, child metrics online calculator program (http://www.childmetrics.com) was used (16).

SH is defined as a serum TSH concentration above the reference range with normal serum fT4 and fT3 levels (11). Euthyroid sick syndrome was defined as low fT3, normal/low fT4 and normal/low TSH (17). TSH, fT3, and fT4 were analyzed by the ‘ECLIA’ chemiluminescence method on a Roche Elecsys E170 (Roche Diagnostics, Indianapolis, IN, USA). TSH, fT4 and fT3 concentrations of the patients included in the study were evaluated by reference to the age range of this kit (18).

### Statistical Analysis

Statistical analyses of the data were performed with SPSS, version 24.0 (IBM Corp., Armonk, New York, USA). The distribution of data was evaluated with the Kolmogorov-Smirnov test. For numerical comparisons, the independent sample t-test or Mann-Whitney U tests were used for parametric and non-parametric distribution of the measured parameters, as appropriate. Categorical data were expressed as frequency (%), while numerical data were expressed as median (25-75^th^ percentile) or mean±standard deviation. In all statistical tests, p values <0.05 were considered significant. Spearman’s rho correlation was used to identify the associations between variables.

## Results

A total of 107 patients with chronic liver disease were included in the study. Of all cases, 53 (49.5%) were female and 54 (50.5%) were male and the median age was 1.25 (interquartile range: 0.28-10.3) years. The median BMI was 15.9 (14.2-17.4) kg/m2 and the mean BMI SDS was -0.80±1.79 (Table 1). The distribution of cases with chronic liver disease is shown in Table 2. Twenty-two patients (19.8%) had chronic liver disease due to congenital metabolic diseases, 14 (13.0%) had chronic viral hepatitis and 34 (31.8%) had cholestatic liver disease.

Of the 107 patients, 96 (89.7%) had normal thyroid function test, seven (6.5%) had SH and four (3.7%) had euthyroid sick syndrome. Of the seven patients with SH, one (14.2%) had GSD, one (14.2%) had biliary atresia, one (14.2%) had undiagnosed cholestatic liver disease, one (14.2%) had Alagille syndrome, one (14.2%) had idiopatic hepatitis, one (14.2%) had progressive familial intrahepatic cholestasis and one (14.2%) had congenital hepatic fibrosis. The distribution of patients with euthyroid sick syndrome was as follows: one patient (25%) had congenital hepatic fibrosis, two patients (50%) had undiagnosed cholestatic liver disease, and one patient (25%) had cryptogenic cirrhosis (Table 3). Correlation coefficients between thyroid function tests and liver function tests are summarized in Table 4. There was a negative correlation between fT3 and direct bilirubin (r=-0.329, p=0.027) and between fT4 and total albumin (r=-0.273, p=0.005). The weight, height and BMI SDS values of patients with abnormal thyroid function tests were significantly lower than those of patients with normal throid function test (p<0.01, p=0.043 and p=0.014, respectively) (Table 5).

## Discussion

In the present study, thyroid function tests were found to be normal in 89.7% of pediatric/adolescent chronic liver disease patients. However, SH was identified in 6.5% and euthyroid sick syndrome in a further 3.7% of these cases with chronic liver diseases. In a study of adult patients conducted by Kharb et al (9) it was reported that 18.7% (14 of 75 cases) with acute and chronic liver disease had thyroid dysfunction and, additionally, 8% (6 of 75 cases) of these patients were reported to have euthyroid sick syndrome. In one study of patients with cirrhosis, the frequence of euthyroid sick syndrome was reported to be 30.6% (10). Experimental studies have shown that the synthesis and release of T4 and T3 are adversely affected by elevated proinflammatory cytokine concentrations (17). In addition, an increase in interleukin 1 beta expression during acute inflammation has been shown to reduce TSH receptor expression (17). The conversion of T4 to T3 was shown to decrease and rT3 concentrations increased, due to decreased deiodinase type 1 activity. Deiodinase type 1 catalyzes both the transformation of serum T4 to active T3 by deiodination of the outer ring and the degradation of T4 to inactive rT3 by deiodination of the outer ring. Type 1 deiodinase is the main enzyme involved in the production of active T3 and as such, reduction in this enzyme activity would contribute to the clinical picture regarding thyroid dysfunction (17). As a result, during acute or chronic inflammation, some cytokines may reduce TSH expression, potentially leading to SH in order to achieve sufficient levels of active thyroid hormone for normal metabolism, and reduce deiodinase type 1 enzyme activity, leading to the biochemical picture of euthyroid sick syndrome (low/normal fT4 and fT3 and elevated rT3).

Clinical management of thyroid dysfunction should be decided according to the presence of possible clinical features of hypothyroidism, the degree of TSH elevation, and changes over time in TSH and free T4 concentrations. In the current study, SH was detected in seven patients. Melis et al (13) investigated pediatric patients with GSD and reported that fT4 concentrations were significantly lower in GSD type 1a and type 1b patients and, moreover, fT3 levels were significantly higher in patients with GSD type 1b than a control group. In the same study, although four of seven patients with GSD type 1b had some degree of hypothyroidism (clinical or subclinical), this was not reported in the patients with GSD type 1a. The present study included 15 cases of GSD and one of them (6.6%) with GSD type 1a, had SH. The prevalence of clinical and SH has been reported to be higher in adult GSD type 1b cases compared to other types (19). It is noteworthy that the rate of detecting thyroid dysfunction in cases with GSD was higher than the other patient groups, which is in accordance with the literature. However, due to the small number of GSD cases in our study, further studies including large case series are needed to confirm if this putative association between is pediatric GSD and thyroid dysfunction is genuine.

Congenital liver disease and hypothyroidism may be manifestations of an underlying genetic defect. For example, *JAG1* gene defect causes Alagille syndrome type 1 and was also tought to be associated with hypothyroidism. In a study that was conducted in 21 Alagille and 100 congenital hypothyroidism patients by de Filippis et al (20), some variants in the *JAG1* gene were found in cases with congenital thyroid defects and unexplained mild hypothyroidism was present in a 28.6% of the Alagille syndrome patients (20). In our study, one case with Alagille syndrome had SH. However, this study was designed as a cross-sectional study and long-term thyroid function test monitoring was not performed. The findings of the de Fillipis et al (20) study suggest that monitoring of thyroid function in patients with Alagille syndrome is warranted.

One (14.2%) of seven patients with SH had biliary atresia. The relationship between biliary atresia and thyroid abnormalities is not yet clear. In a study investigating the genetic etiology of 35 cases with biliary atresia by using single nucleotide polymorphism (SNP) genotyping arrays, heterozygous 2q37.3 deletions was detected in two of these patients, one of whom had congenital hypothyroidism (21). Further studies have shown that 2q37 region contains 18 genes and two of these genes *(ATG4B, ING5)* are highly expressed in thyroid gland except T/B cells (21). Further studies regarding these genetic regions may help us in the elucidation of thyroid hormone abnormalities in patients with biliary atresia.

It was reported that there is a relationship between primary sclerosing cholangitis and autoimmune thyroiditis (22). However, there were no cases of primary sclerosing cholangitis among the patients with cholestasis with SH in our study. Additionally, in the present study, the correlation analysis between thyroid function tests and liver function tests revealed a negative correlation between fT3 and direct bilirubin (r=-0.329, p=0.027). It has been reported that serum fT3 concentrations are associated with the severity of liver function, based on liver function test results. However, there was no data on the relationship between direct bilirubin and fT3 (23). Furthermore, in the present study, weight SDS, height SDS and BMI SDS values of patients with impaired thyroid function tests were found to be significantly lower than those with normal thyroid function test, despite all having chronic liver disease. This could be due to nutritional deterioration, which may be more pronounced in children with thyroid hormone dysfunction, which, in turn, may be related to disease severity or duration. There are a lack of studies regarding this issue and further studies are needed to better define this relationship and also the association between thyroid dysfunction and cholestatic liver diseases.

### Study Limitations

There are some major limitations of our study. These are: i) low number of cases; ii) evaluation based on one thyroid function test measurement and lack of long-term follow-up of thyroid function tests; and iii) ignoring the impact of other drugs on thyroid functions. Moreover, in this retrospective study, since no further investigations such as thyroid antibody testing or thyroid imaging, was performed in patients with thyroid function test abnormalities, no definitive diagnoses could be made in this respect.

## Conclusion

In conclusion, euthyroid sick syndrome or SH may be present in around 10% of children and adolescents with chronic liver diseases. Therefore, thyroid function testing is suggested in these cases at diagnosis and during follow-up. In addition, different types of thyroid dysfunction, according to the types of liver disease, may be seen. Further studies with large case series are needed to better understand the effect of chronic liver disease on thyroid function.

## Figures and Tables

**Table 1:**
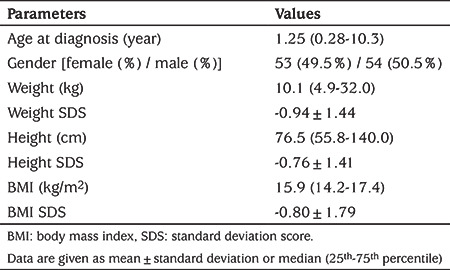
Age, sex distribution and anthropometric characteristics of patients

**Table 2:**
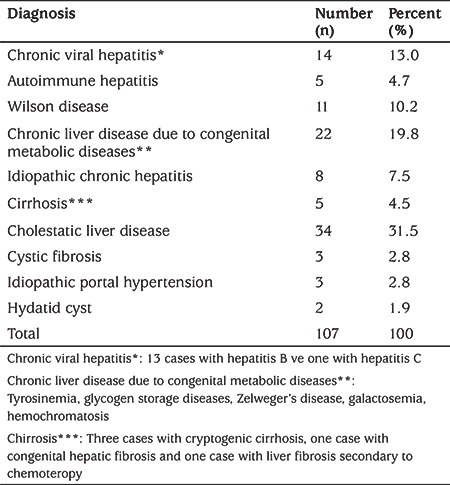
Classification of patients according to their diagnosis

**Table 3:**
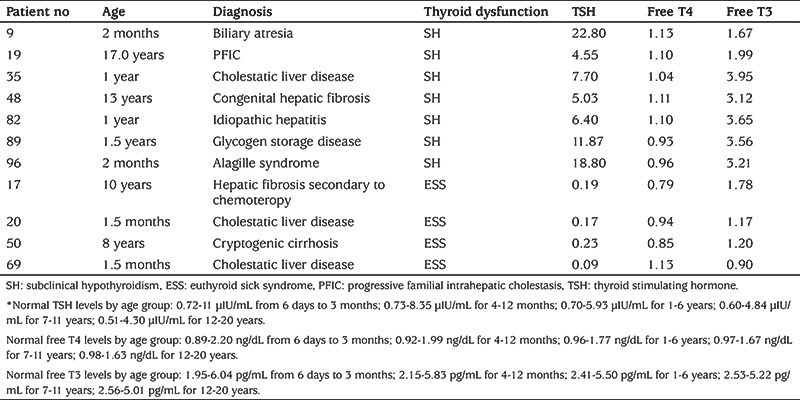
Age, free T3, free T4 and thyroid stimulating hormone levels of patients with thyroid dysfunction*

**Table 4:**
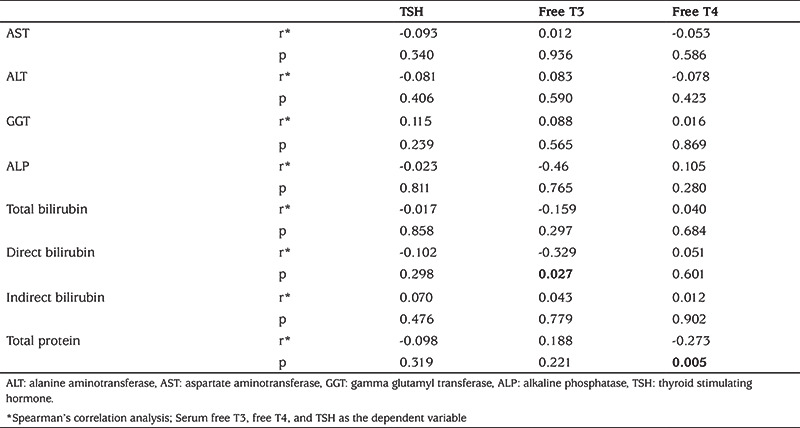
Correlation coefficients between thyroid function tests and liver function tests

**Table 5:**
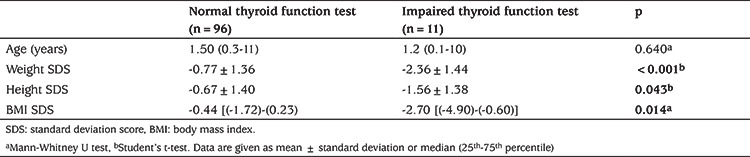
Comparison of body weight height and body mass index standard deviation score of patients with normal and impaired thyroid function tests
